# Prevalence and predictors of postpartum depression and generalized anxiety symptoms among women who delivered at a tertiary hospital in Mwanza Tanzania: a cross-sectional study

**DOI:** 10.1007/s44192-024-00074-5

**Published:** 2024-06-08

**Authors:** Matiko Mwita, Scott Patten, Deborah Dewey

**Affiliations:** 1 Catholic University of Health and Allied Sciences (CUHAS), Mwanza, Tanzania; 2 Psychiatry Department, Bugando Medical Centre (BMC), Mwanza, Tanzania; 3 Departments of Community Health Sciences and Psychiatry, University of Calgary, Calgary, Canada; 4 Departments of Pediatrics and Community Health Sciences, University of Calgary, Calgary, Canada; 5 Department of Psychiatry, Catholic University of Health and Allied Sciences, P.O.Box 1464, Mwanza, Tanzania

**Keywords:** Postpartum women, Postnatal, Mental health, Depression, Anxiety

## Abstract

**Background:**

Postpartum depression and anxiety are major public health concerns that affect 3–39% of women after childbearing and can adversely affect maternal and child health. Most studies have investigated postpartum depression and anxiety and their associated factors among women 4–12 weeks after delivery. There is a scarcity of research among women immediately after delivery from low- and middle-income countries, the gap this study aimed to fill.

**Methods:**

A descriptive cross-sectional study was conducted among 386 postpartum women within one week after delivery. The Edinburg Postnatal Depression Scale was used to assess depressive symptoms and the Generalized Anxiety Disorder − 7 scale was used to screen for symptoms of generalized anxiety disorder. Participants were systematically selected from the postnatal wards and interviewed by trained research assistants from November 2019 to March 2020.

**Results:**

Using standard cut points, the prevalence of depressive and anxiety symptoms was 25.39%, and 37.31% respectively. Having a baby with a weight of 2.5 kgs or more and having partner support were associated with decreased odds of both depression and anxiety symptoms. In contrast, complications during delivery, caesarian section, marital status, and partner violence, were associated with increased odds of depressive and anxiety symptoms post-delivery.

**Conclusion:**

There was a high prevalence of postpartum depression and anxiety symptoms among the study participants in the first week post-delivery, with delivery complications and outcome and psychosocial supports identified as associated factors for depression and anxiety symptoms. These findings highlight the need for early screening to identify those at risk for appropriate intervention.

## Background

Postpartum depression and anxiety are major public health concerns affecting 3–39% of women after childbearing [1], and leading causes of maternal morbidity [2]. Postpartum depression and anxiety refer to depression and anxiety post-delivery [3].They are typically diagnosed 4–12 weeks after childbirth [4] and are associated with adverse effects on maternal and child physical and mental health [5]. Postpartum depression and anxiety have been linked with increased substance use and suicide among breast feeding mothers and negative health-related behaviors and outcomes, including impairments in mother-infant interactions, poor nutrition, inadequate postnatal care, impaired physical development in the infants, and poorer cognitive and social development in the infant [5–10].

Genetic vulnerabilities, hormonal dysregulation, and psychosocial factors have been postulated as risk factors for both postpartum depression and anxiety [11]. The most significant psychosocial factors associated with postnatal depression and anxiety are personal concerns about being a good mother, the health of the newborn, illiteracy, low socio-economic status, unwanted or unplanned pregnancy, stressful life events during pregnancy/puerperium, history of pregnancy or infant loss, low levels of social support, and a poor marital relationship [12–15].

Research that has examined the prevalence of postpartum depression and anxiety has suggested that they are more prevalent in low- and middle-income countries (LMICs) than in high-income countries (HICs) [16]. For postpartum depression, prevalence of 5.2% to 28.8% has been reported in HICs, with most estimates in LMIC ranging from 12.2% to 33% [1, 17]. In sub-Saharan Africa, a study in Kenya reported that 13% of women displayed postpartum depression [18]. Similarly a prevalence rate of 12.2% for postpartum depression was found in a cohort study conducted in Moshi, Tanzania [19]. For postpartum anxiety, a study conducted in Nigeria reported a prevalence rate of 33% [20], while research undertaken in Rwanda reported a prevalence of 31.5% [21]. Finally, a study undertaken in Ethiopia found that 21.2% of the women suffered from both postpartum depression and anxiety [22].

To date, most of the studies that have investigated postpartum depression and anxiety and the factors that predict these disorders has included women 4–12 weeks after delivery. Only a few studies have assessed symptoms of depression and anxiety immediately after delivery and these studies have been conducted in HICs [23–27]. A Korean study that assessed signs of depression one week postpartum using the EPDS reported a prevalence of 8.2% [23]. Another study from United Kingdom assessed symptoms of both depression and anxiety one week postpartum using the EPDS for depression and the State-Trait Anxiety Inventory (STAI) for anxiety. In this study, prevalences of 27.3% and 21.7% was reported for depression and anxiety, respectively [24]. There is a scarcity of research on the symptoms of postpartum depression and anxiety among women immediately after delivery from LMICs and Tanzania, the gap this study aims to fill. Failure to diagnose postpartum depression and anxiety can adversely affect mother’s long term mental health [5] and mother-infant relationships and lead to long-term emotional problems for the child [28]. Early screening and detection are critical for taking action to improve outcomes for the mother and the newborn.

Screening is important, but the performance (in terms of predictive value) of screening instruments for postpartum depression and anxiety depends on the base rates (prevalence) of these conditions in the population screened. Therefore, obtaining this information on symptoms of postpartum depression and anxiety in a population of women in Tanzania immediately after delivery will provide important information to assist in the development of an early screening program that identifies women at risk for postpartum depression and anxiety. Hence, this study aimed to determine the prevalence, and associated factors of depression and generalized anxiety symptoms among women within 1 week post-delivery at Bugando Medical Centre (BMC), Mwanza, Tanzania.

## Materials and methods

### Study design and settings

A cross-sectional study was conducted to determine the prevalence of, and factors associated with, depression and generalized anxiety symptoms among women within one week post-delivery at Bugando Medical Centre, Mwanza, Tanzania. Bugando Medical Centre is a tertiary referral, teaching and research centre for the Lake and Western zones of the United Republic of Tanzania. The hospital has 1000 beds and serves a catchment population of approximately 15 million people; approximately 7200 women deliver at BMC each year [29].

### Participants and recruitment methods

The sample size (N = 386) was recruited from pregnant women who delivered at Bugando Medical Centre between November 2019 and March 2020. A systematic sampling approach was used to recruit participants from the postnatal ward whereby every 2nd woman was approached. Trained research assistants approached the participants, explained the aim of the study, and obtained their consent to participate. For those less than 18 years of age, their assent and parents/guardians’ consent were sought. Potential participants who were physically ill at the time of recruitment were not recruited into the study. Those who scored “high possibility of depression” and above or “moderate anxiety” and above on our measures of depression and anxiety, or had active suicidal behavior were referred to and/or escorted to the psychiatry clinic in BMC for further clinical evaluation and follow-up. Trained research assistants administered the research questionnaires to participants by reading all the questions and recording the participant’s responses. No women dropped out of the study and there was no missing data.

### Research questionnaires

The questionnaires included a sociodemographic and obstetric questionnaire (e.g., age, level of education, marital status, income, occupation, obstetric history, mode of delivery, partner and family support, partner violence), the Edinburg Postnatal Depression Scale (EPDS), and the Generalized Anxiety Disorder − 7 (GAD 7) scale. The EPDS is a 10-item scale that asks women to rate how they felt in the past 7 days. Each question has four possible responses that are scored from 0 to 3. The scores on each of the questions are summed and can range from 0 to 30. A score of 8 and below is considered as depression unlikely, 9–11 depression possible, 12–13 high possibility of depression, 14 and higher probable depression. In this study, participants were classified as displaying symptoms of depression if they obtained a score of nine or above (EDPS) [30].

GAD-7 is a 7-item scale that asks how often the individual has been bothered by specific symptoms associated with anxiety over the past two weeks. Each question has four possible responses, which are scored from 0 to 3. Scores on each question are summed and can range from 0 to 21. A score of 4 and below is classified as no anxiety symptoms, 5–9 mild anxiety, 10–14 moderate anxiety, and 15–21 severe anxiety. Participants were classified as displaying generalized anxiety symptoms if they scored above four on GAD-7 [31].

Both questionnaires are self-reports (though we read all the questions to participants) and have demonstrated good reliability, Cronbach’s α = 0.83 for EPDS [32] and Cronbach’s α = 0.82 for GAD-7 [33]. Both scales have been extensively used with pregnant and postnatal populations and employed in global population-based research [34–37]. They have been used and tested in various countries in Africa and translated into many languages including Swahili, the national language of Tanzania [33, 38, 39].

### Sample size and statistical analysis

To have 80% power to detect a doubling of prevalence in association with an exposure frequency of 21.2% assuming an alpha value of 5%, a sample size of 386 participants was estimated from Kish-Lisle formula of cross sectional studies [22]. Data entry was done using Epi Info software. Data was analyzed using Stata version 15 software for Mac. Categorical variables were summarized using frequencies and percentages, and continuous variables were summarized using means and associated SDs and ranges, or medians and IQR. Descriptive analyses were conducted to examine the sociodemographic characteristics of the sample, and the prevalence and severity of depression and generalized anxiety symptoms. Logistic regressions with 95% CI were used to investigate the associations between sociodemographic and obstetric factors (e.g., age, level of education, marital status, income, occupation obstetric history, mode of delivery and any complication during delivering, partner and family support, partner violence) for postnatal depression and anxiety symptoms, using standard cut-points on the symptom scales. Variables in the univariate analysis were considered for inclusion into the final multivariable logistic model if they had a p < 0.2; the level of significance in the final model was set at p < 0.05.

## Results

### Sociodemographic and obstetric characteristics

The participants ranged in age from 16 to 42 years (M = 27.03; SD = 5.57) and more than two-thirds 71.5% (n = 276) were 16–30 years of age. The mean age of the participant’s partners was 32.8 years (SD = 7.53; range 18 – 72 years). In order to produce interpretable odds ratios these two variables were categorized, see Table 1. Among the study participants, 37.05% (n = 143) had a secondary school level of education, 49.22% (n = 190) were self-employed, and 41.19% (n = 159) had an income of less than 100,000Tshs (45 USD) per month. Most of the participants, 80.83% (n = 312), were married with monogamy being the common type of marriage, 92.31% (n = 288).

**Table 1:** Sociodemographic and obstetric characteristics of the study participants

Variable	Frequency (n)	Percentage (%)
Age of mothers		
16–30	276	71.50
> 30	110	28.50
Age of partners		
18–30	175	45.34
31–40	157	40.67
> 40	54	13.99
Marital status		
Married	312	80.83
Never married	74	19.17
Type of marriage		
Monogamous	362	93.78
Polygamous	24	6.22
Education level		
Never went to school	17	4.40
Primary school	112	29.02
Secondary	143	37.04
College	46	11.92
University	68	17.62
Employment status		
Never employed	108	27.98
Self employed	190	49.22
Employed	88	22.80
Monthly income (Tshs)		
Less than 100,000	159	41.19
100,000–300,000	96	24.87
300,001–500,000	67	17.36
> 500,000	64	16.58
Parity		
Prime gravida	137	35.49
Multiparous	249	64.51
Planned for the pregnancy		
Yes	230	59.59
No	156	40.41
History of pregnancy loss		
Yes	96	24.87
No	290	75.13
History of baby loss		
Yes	53	13.73
No	333	86.27
Mode of delivery		
Spontaneous vagina delivery	241	62.44
Cesarean section	145	37.56
Birth weight of the baby		
< 2.5 kg	60	15.54
≥ 2.5 kg	326	84.46
Did you experience any complication during delivery?		
Yes	99	25.65
No	287	74.35
Does your partner support you in any way?		
Yes	355	91.97
No	31	8.03
Does your family support you in any way?		
Yes	373	96.63
No	13	3.37
Do you experience any form of partner violence?		
Yes	23	5.96
No	363	94.04

Majority of the participants reported being supported by their partner and family, 91.97% and 96.63% respectively; 5.96% of the participants reported experiencing any form of partner violence. Almost two-thirds were multiparous (64.51%; n = 249); and 62.44% (n = 241) of participants delivered by spontaneous vaginal delivery; 25.65% (n = 99) of the participants reported having complications during delivery. Table 1 summarizes the sociodemographic and obstetric characteristics of the study participants.

### Prevalence of depression among women who delivered at BMC

**Fig. 1:**
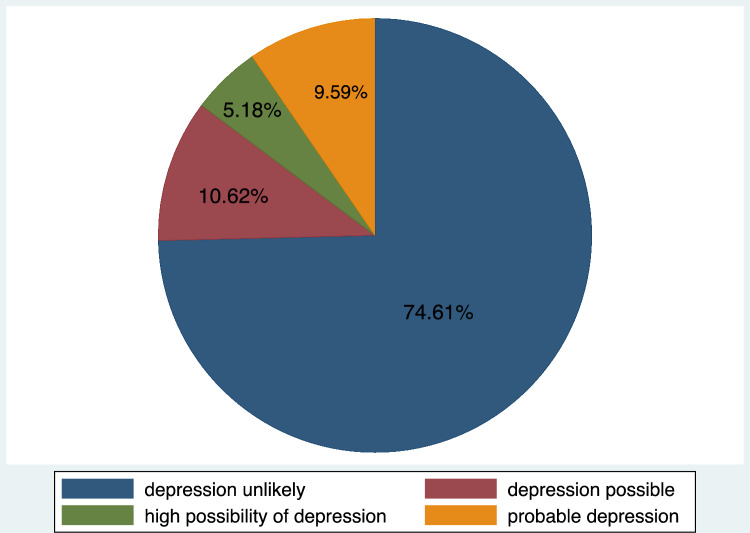
Prevalence and severity of depression symptoms among women who delivered at BMC

Prevalence of depression was classified using scores derived from the Edinburg Postnatal Depression Scale (EPDS). For this study, internal consistency of the EPDS was high; the Cronbach’s α was found to be 0.85. Out of the possible maximum score of 30, the 386 study participants had an average score of 5.27 (SD = 5.49; range = 0 to 23). The prevalence of depression symptoms in this sample was found to be 25.39% (n = 98, 95% CI 21.1%—30.0%). The remaining 74.61% (n = 288) of participants had EPDS scores that were less than nine, which indicated that depression symptoms were unlikely. Figure 1 summarizes the prevalence and severity of depression symptoms among the study participants based on standard cut point scores.

### Prevalence of generalized anxiety symptoms among women who delivered at BMC

**Fig. 2:**
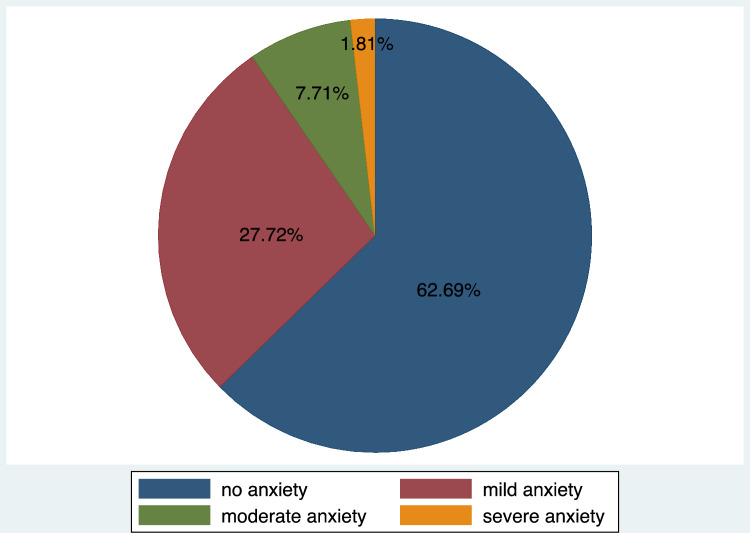
Prevalence of generalized anxiety symptoms among women who delivered at BMC

Prevalence and severity of anxiety was classified using scores derived from Generalized Anxiety Disorder − 7 (GAD 7) scale. For this study, the Cronbach’s α was found to be 0.78, which is acceptable. Out of the possible maximum score of 21, the 386 study participants had an average score of 3.90 (SD = 3.86, range = 0 to 18). The prevalence of anxiety symptoms was found to be 37.31% (n = 144, 95% CI 32.5–42.3%). The remaining 62.69% (n = 242) of participants were classified as not displaying generalized anxiety symptoms based on standard cut point scores. Figure 2 summarizes the prevalence and severity of generalized anxiety symptoms among the study participants based on standard cut point scores.

### Factors associated with depression symptoms

In univariate analysis, marital status, type of marriage, income, planned pregnancy, previous loss of infant, mode of delivery, birthweight, complications at delivery, partner support, family support, and partner violence were significantly associated with depression symptoms (see Table 2). Multivariable analysis (Table 2) revealed that women who were more than 30 years of age (AOR 0.6, 95% CI 0.3, 0.9, p = 0.013), had a university level of education (AOR 0.5, 95% CI 0.2, 0.9, p = 0.025), and an income of more than 500,000/ = Tshs (250 USD) per month (AOR 0.7, 95% CI 0.2, 0.9, p = 0.014) had lower odds of depressive symptoms. Women who were married compared to those who had never married were less likely to report depressive symptoms (AOR 0.7, 95% CI 0.2, 0.8, p = 0.014), and those in polygamous marriages had a 3.7 times greater odds of reporting depressive symptoms (AOR 3.7, 95% CI 1.4, 9.9, p = 0.010). Women who had a planned pregnancy (AOR 0.7, 95% CI 0.4, 0.9, p = 0.017) and those with infants weighing ≥ 2.5 kgs (AOR 0.5, 95% CI 0.3, 0.7, p = 0.016) were less likely to report depressive symptoms. Those who had previously lost a baby (AOR 2.3, 95% CI 1.1, 4.7, p = 0.020) or reported complications during delivery (AOR 1.6, 95% CI 1.9, 2.9, p = 0.019) were significantly more likely to report depression symptoms. Women who delivered by cesarean section had 1.9 times greater odds of depressive symptoms compared to those who delivered by spontaneous vaginal delivery (AOR 1.9, 95% CI 1.1, 3.4, p = 0.022). Women who reported family support (AOR 0.3, 95% CI 0.1, 0.9, p = 0.036) or partner support (AOR 0.9, 95% CI 0.3, 0.6, p = 0.014) were less likely to report depressive symptoms while those who reported partner violence had 5.4 times greater odds of depressive symptoms compared to those who did not report partner violence (AOR 5.495% CI 1.8, 16.4, p = 0.003).

**Table 2:** Associations between sociodemographic and obstetric factors and depression symptoms

Variable	Depression symptoms		Unadjusted OR (95% CI)		Adjusted OR (95% CI)	
	Yes (N %)	No (N %)	OR (95% CI)	P value	OR (95% CI)	P value
Age of mother (years)						
16–30	77 (27.90)	199 (72.10)	1.0		1.0	
> 30	21 (19.09)	89 (80.91)	0.6 (0.4–1.0)	0.074	0.6 (0.3–0.9)	0.013
Age of partner (years)						
18–30	46 (26.29)	129 (73.71)	1.0			
31–40	42 (26.75)	115 (73.25)	1.1 (0.6–1.6)	0.924		
> 40	10 (18.52)	44 (81.48)	0.9 (0.6–1.2)	0.381		
Marital status						
Never married	29 (39.19)	45 (60.81)	1.0		1.0	
Married	69 (22.12)	243 (77.88)	0.4 (0.3–0.8)	0.003	0.7 (0.2–0.8)	0.014
Type of marriage						
Monogamous	87 (24.03)	275 (75.97)	1.0		1.0	
Polygamous	11 (45.83)	13 (54.17)	2.7 (1.2–6.2)	0.021	3.7 (1.4–9.9)	0.010
Education level						
Never went to school	2 (11.76)	15 (88.24)	1.0		1.0	
Primary	31 (27.68)	81 (72.32)	2.9 (0.6–13.3)	0.177	4.8 (0.9–27.2)	0.078
Secondary	36 (25.17)	107 (74.83)	2.5 (0.6–11.6)	0.234	5.2 (0.9–31.1)	0.071
College	12 (26.09)	34 (73.91)	2.6 (0.5–13.3)	0.238	6.6 (0.9–45.9)	0.055
University	17 (25.00)	51 (75.00)	2.5 (0.5–12.1)	0.254	0.5 (0.2–0.9)	0.025
Employment						
Never employed	33 (30.56)	75 (69.44)	1.0		1.0	
Self employed	45 (23.68)	145 (76.32)	0.7 (0.4–1.2)	0.196	1.2 (0.5–2.9)	0.657
Employed	20 (22.73)	68 (77.27)	0.6 (0.3–1.3)	0.221	1.2 (0.4–3.8)	0.755
Monthly income (Tshs)						
< 100,000	49 (30.82)	110 (69.10)	1.0		1.0	
100,001–300,000	18 (18.75)	78 (81.25)	0.5 (0.3–0.9)	0.036	0.5 (0.2–1.2)	0.125
300,001–500,000	18 (26.87)	49 (73.13)	0.8 (0.4–1.5)	0.553	0.9 (0.3–2.5)	0.836
> 500,000	13 (20.31)	51 (79.69)	0.6 (0.3–1.1)	0.116	0.7 (0.2–0.9)	0.014
Parity						
Prime	35 (25.55)	102 (74.45)	1.0			
Multiparous	63 (25.30)	186 (74.70)	0.9 (0.6–1.6)	0.958		
Planned for the pregnancy						
No	52 (33.33)	104 (66.67)	1.0		1.0	
Yes	46 (20.0)	184 (80.0)	0.5 (0.3–0.7)	0.003	0.7 (0.4–0.9)	0.017
History of pregnancy loss						
No	72 (24.83)	218 (75.17)	1.0			
Yes	26 (27.08)	70 (72.92)	1.1 (0.6–1.8)	0.660		
History of baby loss						
No	76 (22.82)	257 (77.18)	1.0		1.0	
Yes	22 (41.51)	31 (58.49)	2.3 (1.3–4.3)	0.004	2.3 (1.1–4.7)	0.020
Mode of delivery						
Spontaneous vaginal delivery	49 (20.33)	192 (79.67)	1.0		1.0	
Caesarian Section	49 (33.79)	96 (66.21)	2.0 (1.2–3.2)	0.004	1.9 (1.1–3.4)	0.022
Baby birth weight						
< 2.5	24 (40.0)	36 (60.0)	1.0		1.0	
≥ 2.5	74 (22.70)	252 (77.30)	0.4 (0.3–0.8)	0.005	0.5 (0.3–0.7)	0.016
Did you experience any complication during delivery?						
No	60 (20.91)	227 (79.09)	1.0		1.0	
Yes	38 (38.38)	61 (61.62)	2.4 (1.4–3.9)	0.001	1.6 (1.9–2.9)	0.019
Does your partner support you in any way?						
No	15 (48.39)	16 (51.61)	1.0		1.0	
Yes	83 (23.38)	272 (76.62)	0.3 (0.2–0.6)	0.003	0.9 (0.3–0.6)	0.014
Does your family support you in any way?						
No	7 (53.85)	6 (46.15)	1.0		1.0	
Yes	91 (24.40)	282 (75.60)	0.3 (0.1–0.8)	0.024	0.3 (0.1–0.9)	0.036
Do you experience any form of partner violence?						
No	83 (22.87)	280 (77.13)	1.0		1.0	
Yes	15 (65.22)	8 (34.78)	6.3 (2.6–15.4)	<0.001	5.4 (1.8–16.1)	0.003

### Factors associated with anxiety symptoms

In univariate analysis, monthly income, mode of delivery, birth weight, complications during delivery, partner support, and partner violence were significantly associated with anxiety symptoms (see Table 2). In multivariable analysis (Table 2), women whose partners were between 31–40 years of age had a 1.9 times greater odds of reporting anxiety symptoms (AOR 1.9, 95% CI 1.1, 3.1, p = 0.019). Women who had complications during delivery had 1.4 times greater odds (AOR 1.4, 95% CI 1.8, 2.3, p = 0.012) of developing anxiety symptoms, and those who delivered by caesarian section had a 1.7 times greater odds of developing anxiety symptoms (AOR 1.7, 95% CI 1.1, 2.8, p = 0.028). Women with university level of education had 50% less likelihood of reporting anxiety symptoms (AOR 0.5, 95% CI 0.6, 0.9, p = 0.042). Women who gave birth to babies who had birthweights of 2.5 or greater were less likely to report anxiety symptoms (AOR 0.6, 95% CI 0.3, 0.9, p = 0.012). Women who reported partner support were significantly less likely to have anxiety symptoms (AOR 0.5, 95% CI 0.2, 0.5, p = 0.017), while those who reported partner violence had a 4.2 times greater odds of reporting anxiety symptoms (AOR 4.2, 95% CI 1.2, 2.7, p = 0.039) compared to those who did not report partner violence. Table 3 summarizes the associations between sociodemographic and obstetric factors and anxiety symptoms among study participants.

**Table 3:** The associations between sociodemographic and obstetric factors and anxiety symptoms

Variable	Anxiety		Univariate OR (95% CI)		Multivariate OR (95% CI)	
	Yes (N %)	No (N %)	OR (95% CI)	P value	OR (95%CI)	P value
Age mother (years)						
16–30	107 (38.77)	169 (61.23)	1			
> 30	37 (33.64)	73 (66.36)	0.8 (0.5–1.3)	0.347		
Age partner (years)						
18–30	59 (33.71)	116 (66.29)	1.0		1.0	
31–40	66 (42.04)	91 (57.96)	1.4 (0.9–2.2)	0.119	1.9 (1.1–3.1)	0.019
> 40	19 (35.19)	35 (64.81)	1.1 (0.6–2.0)	0.842	1.5 (0.7–3.2)	0.242
Marital status						
Never married	32(43.24)	42(56.76)	1.0			
Married	112(35.9)	200(64.10)	0.7(0.4–1.2)	0.241		
Type of marriage						
Monogamous	135(37.29)	227(62.71)	1.0			
Polygamous	9(37.50)	15(62.50)	1.0(0.4–2.4)	0.984		
Education level						
Never went to school	3 (17.65)	14 (82.35)	1.0		1.0	
Primary	42 (37.50)	70 (62.50)	2.8 (0.8–10.3)	0.122	2.9 (0.7–11.4)	0.123
Secondary	60 (41.96)	83 (58.04)	3.3 (0.9–12.3)	0.065	4.8 (1.0–19.4)	0.064
College	15 (32.61)	31 (67.39)	2.3 (0.6–9.0)	0.251	4.1 (0.8–18.8)	0.072
University	24 (35.29)	44 (64.71)	2.5 (0.7–9.7)	0.173	0.5 (0.6–0.9)	0.042
Employment Status						
Housewife	39 (36.11)	69 (63.89)	1.0			
Self-employment	80 (42.11)	110 (57.89)	1.3 (0.8–2.1)	0.310		
Employed	25 (28.41)	63 (71.59)	0.7 (0.4–1.3)	0.254		
Monthly income (Tshs)						
< 100,000	64 (40.25)	95 (59.75)	1.0		1.0	
100,001–300,000	33 (34.38)	63 (65.62)	0.8 (0.5–1.3)	0.349	0.6 (0.3–1.1)	0.120
300,001–500,000	32 (47.76)	35 (52.24)	1.4 (0.8–2.4)	0.298	1.0 (0.5–2.1)	0.948
> 500,000	15 (23.44)	4 9(76.56)	0.5 (0.2–0.9)	0.019	0.3 (0.1–1.2)	0.129
Parity						
Prime	46 (33.58)	91 (66.42)	1.0			
Multiparous	98 (39.36)	151 (60.64)	1.3 (0.8–2.0)	0.262		
Planned for the pregnancy						
No	6 2(39.74)	94 (60.26)	1.0			
Yes	82 (35.65)	148 (64.35)	0.8 (0.6–1.3)	0.415		
History of pregnancy loss						
No	106 (36.55)	184 (63.45)	1.0			
Yes	38 (39.58)	58 (60.42)	1.1 (0.7–1.8)	0.595		
History of baby loss						
No	123 (36.94)	210 (63.06)	1.0			
Yes	21 (39.62)	32 (60.38)	1.1 (0.6–2.0)	0.707		
Mode of delivery						
Spontaneous vaginal delivery	76 (31.54)	165 (68.46)	1.0		1.0	
Caesarian Section	68 (46.90)	77 (53.10)	1.9 (1.3–2.9)	0.003	1.7 (1.1–2.8)	0.028
Birth weight of the baby						
< 2.5	32 (53.33)	28 (46.67)	1.0		1.0	
≥ 2.5	112 (34.36)	214 (65.64)	0.5 (0.3–0.8)	0.006	0.6 (0.3–0.9)	0.012
Did you experience any complications during delivery?						
No	98 (34.15)	189 (65.85)	1.0		1.0	
Yes	46 (46.60)	5 3(53.53)	1.7 (1.1–2.6)	0.030	1.4 (1.8–2.3)	0.012
Does your partner support you in any way?						
No	18 (58.06)	13(41.94)	1.0		1.0	
Yes	126 (35.49)	229 (64.51)	0.4 (0.2–0.8)	0.015	0.5 (0.2–0.5)	0.017
Does your family support you in any way?						
No	6 (46.15)	7 (53.85)	1.0			
Yes	138 (37.00)	235 (63.00)	0.6 (0.2–2.0)	0.504		
Do you experience any form of partner violence?						
No	128 (35.26)	235 (64.74)	1.0		1.0	
Yes	16 (69.57)	7 (30.43)	4.2 (1.7–10.4)	0.002	4.2 (1.2–2.7)	0.039

## Discussion

The findings from this study revealed that symptoms of depression were prevalent in 25% of postpartum women in the first week after the birth of their infant. This was lower than that reported in a study of US military women where postpartum depression assessed immediately after delivery was reported in almost a half of the participants [25]. However, the prevalence of depression among the women in our study was higher than that found in studies conducted in HICs among women 1 week post-delivery; a study conducted in Korea in 2010 reported a prevalence of 8.2% [23], while a study conducted in Sweden in 2012 reported a prevalence of 9.4% [26] and a study conducted in Italy in 2007 reported a prevalence of 15.7% [27]. The prevalence found in the present study was similar to that observed in a study conducted in the UK by Jassen et al. in 2018 where a prevalence of depressive symptoms of 27.3% was reported one week post-delivery [24]. We also found that anxiety symptoms were prevalent in 37.3% of our participants within one week post-delivery. This was much higher than that reported by Jassen et al. (i.e., 21.7%) in a study conducted in the UK [24]. The discrepancies among studies in the prevalence of both depression and generalized anxiety symptoms could be due to sociodemographic and socio-cultural differences in study participants, differences in the measures used to assess depression and anxiety symptoms (standard clinical interviews versus screening tool), and different cut-off scores used on the screening measures to classify participants as displaying depression and generalized anxiety [40, 41]. The relatively high frequency of depression and anxiety among the women in our study who were screened within one week after delivery supports the potential value of early screening. In settings where formal evaluation is not viable, the results of early screening could be used to help identify those at highest risk. These women could be targeted for early interventions that could reduce the risk of these women developing more severe mental health concerns and result in improved maternal and child health outcomes.

In this study, women who were married, were less likely to develop symptoms of postpartum depression as compared to those who were single. This is consistent with the findings of a systematic review conducted in 2001 that examined predictors of postpartum depression. It reported that women who were married had reduced odds for symptoms of postpartum depression [42]. Single mothers may experience a number of psychosocial disadvantages that may lead to stressful life situations and alter psychosocial stability, which may increase the risk of postpartum depression [43]. Even though being married may be protective against developing postpartum depression, type of marriage can play a significant role as noted in the present study. Those in a polygamous marriage were significantly more likely to report depression. This has been observed in previous studies conducted in Sub-Saharan Africa [44].

In this study maternal age and planned pregnancy were found to be associated with depressive symptoms. This is consistent with findings reported by Silverman et al., who reported that younger the age and/or unplanned pregnancy were associated with higher the odds for depressive symptoms [45]. We also found that level of education was significantly associated with both depressive and anxiety symptoms, which is similar to previous research [12, 15].

Obstetric complications were associated with postpartum depression and anxiety. Specifically, we found that complications during delivery and type of delivery were factors associated with both postpartum depression and anxiety. Further, a previous history of infant loss was also associated with postpartum depression. Similar findings have been reported in previous studies. Silverman et al. reported complications during delivery such as obstructed labour or having a preterm birth increased the odds for depressive symptoms [45], Gaillard et al. reported that mode of delivery, specifically cesarean section or instrumental assistance mode of delivery, were associated with increased odds for postpartum depression [46]. Prokopowicz et al. found that cesarean section mode of delivery and previous pregnancy loss were associated with anxiety symptoms [47]. These findings indicate that a negative obstetric history or losing their index baby. are associated with an increased risk of postpartum depression or generalized anxiety in the week after delivery and support the importance of obstetric history as a predictor for perinatal mental health.

This study also found a strong association between family and partner support and maternal depression and anxiety during the postnatal period. Those with family or partner support were less likely to develop depression or anxiety symptoms compared to those with no family or partner support. These findings highlight the importance of partner and family support in the Tanzanian context and are consistent with evidence from previous studies in Ethiopia [48] and Tanzania [49] that have examined women’s emotional status in the postpartum period. Partner violence plays a significant role in postpartum depression and anxiety, and previous studies have reported that intimate partner violence, particularly physical violence was associated with increased odds for both postpartum depression and anxiety symptoms [14]. The association between partner violence and postnatal mental disorders in women is well documented in HICs [17] and growing data is emerging from LMICs countries that supports this association [50, 51]. These findings emphasize the need for the public, practitioners, and authorities to be aware of the mental health consequences that can follow from partner violence during perinatal period. Psychological support is needed in conjunction with community and government interventions to reduce partner violence.

## Strengths

We used valid and reliable questionnaires to collect data on symptoms of depression and anxiety from a large sample of women who delivered in a larger tertiary hospital in Tanzania within one week after delivery to determine prevalence. We also examined several relevant predictors of symptoms of depression and anxiety.

## Limitations

A cross sectional methodology was used, which relied on self-report of symptoms. This could be associated with recall bias, that is under or over reporting of the symptoms. Women were interviewed by research assistants, which could result in reporting bias and social desirability bias, which could result in the women reporting fewer symptoms than they actually experience. Therefore, the results obtained could be an underestimation of prevalence. The study was done at the largest tertiary hospital in the Lake and Western Zone of Tanzania, which serves a diverse population with regional difference; however, it’s also the referral centre for women with high-risk obstetrics histories, which would differentiate the population who deliver here from those who deliver in the community; these women may be more at risk of experiencing concerns regarding their pregnancy outcomes and may be at higher risk of depression and anxiety.

## Conclusion

The prevalence of postpartum depression and anxiety symptoms among women in the first week after delivery at BMC in Mwanza, Tanzania was relatively high with a quarter of women screening positive for depressive symptoms and over one third screening positive for generalized anxiety disorder symptoms. Delivery complications, pregnancy outcome, marital status, psychosocial support network, and intimate partner violence were identified as factors associated with postpartum depression and anxiety symptoms in this population. Postpartum depression and anxiety can have significant health consequences to the mother and child. These findings support the need for early screening and detection of those at risk for postpartum depression and generalized anxiety so that appropriate intervention can be provided to reduce potential long-term morbidity and disability. The associations reported here will assist with the identification of high-risk groups, which may be helpful for the targeting screening efforts, interpreting the results of screening assessments, and identifying opportunities for preventions.

Findings from this study are a call to policy makers, health care planners, and the government to develop a better understanding of the magnitude of postpartum common mental disorders and to investigate outcomes associated with early diagnosis and treatment. It provides strong evidence supporting the importance of integrating mental health services into existing antenatal and postnatal care services.

## Data Availability

The datasets used and/or analysed during the current study are available from the corresponding author on reasonable request.
